# Report of Some of the Proceedings of the American Medical Association

**Published:** 1881-06

**Authors:** 


### REPORT OF SOME OF THE PROCEEDINGS OF THE AMERICAN MEDICAL ASSOCIATION.

At the last Annual Meeting of the American Medical Association, which convened at Richmond, Va., on May 3d, 1881, an important advance step was taken by that body in establishing a section on dentistry. Among the dentists present were Dr. Jacob L. Williams, of Mass., Dr. D. H. Goodwillie, of New York, D. C. Hawxhurst, of Mich., Parmly, of Conn., E. S. Talbot, T. W. Brophy and W. W. Allport, of Chicago. Others were expected and had promised to be present, but, influenced no doubt, by the fear that the Association would turn a cold shoulder to the movement, they failed to put in an apppearance.

During the first and second days of the session the matter was talked over with such prominent members of the profession as Professors Gross, of Phila., Davis of Chicago, Sayre of New York, Dunster of Ann Arbor, Stone of St. Paul, and others. Without exception the suggestion met with most cordial approval and all the assistance which they could render was generously proffered. On the third morning of the session, just after the reading of the minutes, Professor Gross stepped upon the platform, and, in his usual impressive manner, moved for the suspension of the regular order of business, "with a view," as he said, "of offering an amendment to one of the by-laws, contemplating the formation of a new section to be known as the "Section on Dentistry."

In support of this amendment, Professor Gross said: "When we consider, Mr. President, the value of dentistry and the fact that it is universally regarded as one of the necessities of civilized life, it is surprising that such a section was not established long ago. Dentistry is the oldest of all the specialties. It has been practiced from time immemorial, and in modern times has acquired a degree of importance not exceeded by any one branch of the healing art. There are at this moment not fewer than fourteen or fifteen dental colleges in the United States alone, and thousands of educated dentists in the enjoyment of a successful practice.

"Dentistry has a copious literature; many journals are published in its interest, and numerous associations attest the ties by which its members are cemented together. If these things be true—and I vouch for their accuracy—it is, it seems to me, eminently proper that this Association should, without delay, form a section such as is contemplated by my motion. The Association has long had a section on opthalmology, one on otology and one on laryngology; and surely, if these specialties are entitled to such a distinction, it is not difficult to find reasons for placing dentistry upon a similar footing. Every man, woman and child in the civilized world requires the services of the dentist; whereas, comparatively few persons require the services of the oculist, the aurist, or the laryngologist.

"The claims of dentistry, when properly practiced, to an elevated professional status, are everywhere recognized, while the social status of the dentist is steadily progressive. If dentistry was once simply an art, it is now an art and a science—practiced in all enlightened communities by educated and refined men. I hope therefore that the sanction of the house will be given to my motion."

Professor Sayre, of New York, also being upon the platform, arose and said;—"This motion meets with my cordial approval, and I second it with all my heart."

Professor Davis, of Chicago, also seconded the motion with the following very appropriate remarks:—"Oral, or dental surgery is as much a legitimate department of medicine as Opthalmology, Otology or Gynecology, and as there are at present enough members of the Association who are practicing dentistry to commence a good section, I hope the proposition to create one will be cordially sustained by the Association. Its practical effect will be to increase the number of fully educated dentists and by bringing them into closer relations with the great body of the profession, greatly advance the mutual interests of all parties."

The motion being thus warmly urged by these veteran members of the profession and ex-presidents of the Association, it was adopted without a dissenting vote.

On the fourth day the Association designated Dr. Goodwillie of New York, as Chairman and Dr. Brophy of Chicago, as Secretary of the Section; but in conformity with one of the by-laws, all work by the section is postponed for one year.

Thus it will be seen, that the long-hoped for, but never expected recognition of the treatment of the teeth and the diseases incident thereto, as a special department in medical practice, has been established by the assembled representatives of the medical profession in the United States. And in the future, all practitioners of dental surgery holding a medical degree, will, with proper credentials, be admitted to membership in the American Medical Association upon an equal footing with the practitioners of any other department of medicine: and the year 1881 marks an important era in the history of dentistry.

